# Comparison and evaluation of lupus nephritis response criteria in lupus activity indices and clinical trials

**DOI:** 10.1186/s13075-015-0621-6

**Published:** 2015-04-28

**Authors:** Kristin M Corapi, Mary Anne Dooley, William F Pendergraft

**Affiliations:** Division of Nephrology, Department of Medicine, Massachusetts General Hospital, 55 Fruit St., Boston, MA 02114 USA; Division of Nephrology, Department of Medicine, University of North Carolina (UNC) Kidney Center, 7024 Burnett-Womack Building, Campus Box # 7155, Chapel Hill, NC 27599-7155 USA

## Abstract

Systemic lupus erythematosus (SLE) is a systemic autoimmune disease with diverse manifestations. Although the approval of new therapies includes only one agent in 50 years, a number of promising new drugs are in development. Lupus nephritis is a dreaded complication of SLE as it is associated with significant morbidity and mortality. Advancing the treatment of lupus nephritis requires well-designed clinical trials and this can be challenging in SLE. The major obstacles involve identifying the correct population of patients to enroll and ensuring that a clinically appropriate and patient-centered endpoint is being measured. In this review, we will first discuss the clinical utility of endpoints chosen to represent lupus nephritis in global disease activity scales. Second, we will review completed and active trials focused on lupus nephritis and discuss the endpoints chosen. There are many important lessons to be learned from existing assessment tools and clinical trials. Reviewing these points will help ensure that future efforts will yield meaningful disease activity measures and well-designed clinical trials to advance our understanding of lupus management.

## Introduction

Kidney involvement in lupus, particularly in high-risk populations, can lead to end-stage kidney disease (ESKD). Carefully designed trials to identify strategies to calm flares of lupus nephritis (LN) and identify drug regimens to maintain remission are essential. Historically, high-dose corticosteroids were the mainstay of treatment for LN, and not until the mid-1980s was cyclophosphamide found to prolong renal survival [1]. Subsequently, glucocorticoid therapy and cyclophosphamide became the standard induction regimen. Research has focused on identifying regimens that allow shorter treatment courses, lower glucocorticoid doses, and less toxicity [2]. No therapies are approved for the treatment of systemic lupus erythematosus (SLE) aside from aspirin, prednisone, hydroxychloroquine, and belimumab. In this review, we will focus on previous and ongoing trials specifically related to the treatment of LN.

Critical analysis of completed LN clinical trials will allow us to design effective and meaningful clinical trials in the future. A well-designed study requires precise inclusion and exclusion criteria, guidelines on standardized steroid dosing, and carefully chosen endpoints. SLE is a systemic disease, and although a particular treatment might benefit kidney involvement, an awareness of the impact on disease activity in other organs is essential. Numerous global disease activity indices have been developed to quantify disease activity. In this review, we will evaluate the renal response criteria within the most frequently used disease activity indices and determine whether they can be translated easily into clinical practice. We will also evaluate the clinical utility of endpoints in past and current clinical trials in LN.

## Lupus nephritis disease burden

SLE is a complex and potentially life-threatening autoimmune disease. Kidney involvement, which affects the majority of patients, remains the most significant cause of morbid and mortal complications [3,4]. The incidence of ESKD and the overall mortality among patients with LN have not improved in populations studied in London, Toronto, and the United States [5]. In fact, United States Renal Data System data analysis of trends in outcomes of ESKD due to incident LN from 1995 to 2006 revealed that the incidence of ESKD is increasing [6]. In addition, renal flares may be an independent predictor of incident and progressive chronic kidney disease (CKD) [7]. Patients with lower socioeconomic status have an even worse prognosis [8-10]. Advances in identification and characterization of etiologic and pathogenic mechanisms underlying LN have not led to more effective treatments for LN, despite advances in the treatment of important co-morbidities, including diabetes, hyperlipidemia, and hypertension [4,11,12]. A sobering fact remains: up to 70% of patients with LN are resistant to current immunosuppressive regimens [13]. Filling this gap is paramount for the prevention, treatment, and cure of LN. The current clinical management of patients with LN remains limited to the use of non-specific cytotoxic drugs despite the advent of numerous potential biologic agents [3].

## Surveillance of lupus nephritis in clinical nephrology

Kidney involvement in SLE is heralded by either the presence of proteinuria (>0.5 g/day), active urinary sediment (with red blood cell, granular, tubular and/or mixed casts), or an unexplained rise in serum creatinine. A kidney biopsy is the gold standard to diagnose LN as it provides information regarding the pattern and severity of renal involvement as well as the stage, activity, and chronicity. These are all important considerations influencing treatment decisions [14]. Immunosuppressant medication is used to halt kidney injury when proliferative disease is found at biopsy. The pathologic classification of LN has evolved—the most recent International Society of Nephrology/Renal Pathology Society 2003 classification of LN guidelines was published in 2004—in an effort to better capture the spectrum of kidney involvement [15].

Nephrologists monitor LN activity by trending the estimated glomerular filtration rate (eGFR) and proteinuria and by conducting an interval examination of the urine sediment. There are several techniques used to determine eGFR: the Modification of Diet in Renal Disease, Cockcroft-Gault, or CKD-epi equations. There is no consensus as to which estimating equation is preferred, and head-to-head comparisons are inconclusive. Each estimating equation has advantages and disadvantages in certain clinical settings. Another option is to measure the GFR by using a 24-hour urine collection; however, this is burdensome for the patient and open to technique problems such as under-collection [15-17].

Just as no consensus exists to the best method of determining GFR, there is more than one approach to determining the quantity of proteinuria. Some centers use spot urine samples to calculate the urine protein-to-creatinine ratio, whereas others opt for a 24-hour or timed sample to quantify protein excretion. Recent work suggests that the spot protein-to-creatinine ratio may be inaccurate in the assessment of the degree of proteinuria in LN as compared with other forms of chronic glomerular disease; however, prospective studies are needed to confirm and validate this finding [16].

Finally, serial urinalyses to detect hematuria and re-examination of the sediment to look for cellular or mixed casts also help the treating physician determine whether active kidney involvement persists. An assessment of eGFR, proteinuria, and urinary sediment is essential to the early detection of LN flares and to allow prompt intervention. A standardized approach to each facet of LN surveillance is needed to allow comparisons of treatment strategies.

## Renal subscales in systemic lupus erythematosus disease activity scales

Given the systemic nature of SLE, it is important to monitor overall lupus disease activity when instituting therapy. A treatment may benefit one organ system at the expense of worsening symptoms in another. Previous guidance from the US Food and Drug Administration (FDA) suggests that clinical trials in SLE be designed with a primary endpoint of overall disease activity [17]. Table 1 summarizes the renal subscales of some of the commonly used lupus activity instruments [18]. There are additional disease activity scales, but not all include a renal subscale [19,20]. In general, the disease activity scales include various SLE manifestations and the clinician is asked to ascertain the presence or absence of each by using the definitions supplied and then to establish the disease activity score.

**Table 1:** **Renal response criteria in global systemic lupus erythematosus scoring tools**

**Scale**	**Time period**	**Scoring**	**Renal response criteria**	**Strengths and criticisms [** 18 **]**
Disease activity				
SLEDAI-2K [45]	10 days	• The presence of each of the renal manifestations adds 4 points to patient’s total score.	• Urinary casts (heme-granular or RBC)	✓ Validated for clinical and research use
			• Hematuria (>5 RBCs/hpf)	○ Does not capture improving or worsening
			• Proteinuria (>0.5 g/24 hours, new onset or increase of >0.5 g/24 hours)	○ Must wait for labs to score
			• Pyuria (>5 WBCs/hpf, excluding infection)	
BILAG [46]	One month	• Category A (severe disease) = ≥2 of the following:	• Blood pressure	✓ Incorporates an element of change
		1. Proteinuria, defined as	• Accelerated hypertension?	✓ Sensitive to small changes
		(a) urinary dipstick increased by 2 or more levels	• Proteinuria (on either dipstick or 24-hour collection)	✓ Can identify if disease improving, stable, or worse
		(b) 24 urinary protein rising from <0.20 to >1 g	○ >1 g/24 hours?	± scoring can be complex (computer program available)
		(c) 24 hour urinary protein rising from >1 g by 100%	○ UPCR >100 mg/mmol?	
		(d) newly documented proteinuria of >1 g	• Nephrotic syndrome?	○ Requires formal training
		2. Accelerated hypertension	• Creatinine	○ Developed for research
		3. Deteriorating kidney function, defined as	• Creatinine clearance/GFR	○ Up to 50 minutes to complete
		(a) plasma creatinine >130 μmol/L and having risen to >130%	• Active urinary sediment (>5 WBCs/hfp, >5 RBCs/hpf, or RBC casts)	○ Must wait for labs to score
		(b) creatinine clearance fallen to <67% of previous value	• Histologic evidence of nephritis in the previous 3 months? (excludes sclerosis)	
		(c) creatinine clearance <50 mL/min, and last time was >50 mL/min or was not measured		
		4. Active urinary sediment		
		5. Histological evidence of active nephritis		
		• Category B (moderate disease) = one of the following:		
		1. Any one of the category A criteria above		
		2. Proteinuria		
		(a) urinary dipstick which has risen by 1+ or >2+		
		(b) 24-hour urinary protein rising from >1 g by >50% but <100%		
		3. Plasma creatinine >130 μmol/L and having risen 115%		
ECLAM	One month	• 0.5 points for each renal criteria present	• Proteinuria ≥500 mg/day	✓ Derived from a large number of real patients and standardized data
		• 2 extra points added if only kidney involvement	• Urinary casts (RBCs, hemoglobin, granular, tubular, or mixed)	✓ Easy to administer and scor
		• 2 points for evolving manifestations (if any renal symptom new or worse since last evaluation)	• Hematuria (micro- or macro-scopic)	○ Global score will miss changes in severity over time
			• Raised serum creatinine or reduced creatinine clearance	
SLAM-R	One month	• Hypertension (diastolic pressure, mm Hg)	No specific renal response criteria but the following components are renal-related:	✓ Evaluates activity and severity
		- 0: <90	• Hypertension	✓ Computerized version available
		- 1: 90-104	• Raised serum creatinine or reduced creatinine clearance	○ Lacks immunologic markers
		- 2: 105-114	• Severity of urine sediment analysis per high-power field	○ Not used in major ongoing clinical trials
		- 3: >115		
		- Unknown		
		• Serum creatinine or creatinine clearance (% normal)		
		- 0: 0.5-1.0 or 80%-100%		
		- 1: 1.4-2.0 or 60%-79%		
		- 2: 2.1-4.0 or 30%-59%		
		- 3: >4.0 or <30%		
		- Not recorded		
		• Urine sediment		
		- 0: normal		
		- 1: 6-10 RBCs or WBCs OR 0-3 granular or non-RBC casts OR trace-1+ protein (<500 mg/L 24-hour urine protein)		
		- 2: 11-25 RBCs or WBCs OR >3 granular or non-RBC casts OR 2-3+ protein (>500 mg to 3.5 g/L 24-hour urine protein)		
		- 3: >25 RBCs or WBCs OR any RBC casts OR 4+ protein (>3.5 g/L 24-hour urine protein)		
Disease damage				
SLICC/ACR Damage Index [47]	Cumulative damage index	1 point for satisfying GFR or proteinuria	• Estimated or measured GFR <50%	✓ Ability to assess accumulated damage
		3 points if ESKD	• Proteinuria ≥3.5 g/24 hours	✓ Prognostic tool
			• ESKD	○ Recommended for clinical trials to describe population
				○ Accuracy depends on information available

BILAG, British Isles Lupus Assessment Group Index; ECLAM, European Consensus Lupus Activity Measurement; ESKD, end-stage kidney disease; GFR, glomerular filtration rate; hpf, high-power field; RBC, red blood cell; SLAM-R, Systemic Lupus Activity measure-revised; SLEDAI-2 K, systemic lupus erythematosus Disease activity index- 2K; SLICC/ACR, Systemic Lupus International Collaborating Clinics/American College of Rheumatology; UPCR, urinary protein-to-creatinine ratio; WBC, white blood cell.

Disease activity indices that include surveillance parameters routinely assessed in clinical practice are more easily translated into clinical use as the physician already has the necessary data. eGFR and proteinuria are both objective and routinely measured by treating physicians. As discussed earlier, recent evidence suggests that a 24-hour collection is more reliable than a spot urine sample to quantify proteinuria in LN, albeit much more difficult for patients to perform [16]. eGFR can be estimated from one of many equations, but prospective work is needed to determine the most accurate and reliable equation in LN. These two measures of kidney function are routinely performed as part of clinical care and are easy to interpret; thus, their inclusion as endpoints in renal subscales makes for a seamless transition from a research setting to the clinic. We feel strongly that any assessment of LN activity should include a measure of both proteinuria and glomerular filtration.

Although the urine sediment gives important clues to the presence of ongoing nephritis, slide preparation and interpretation are operator-dependent. Benign kidney disease such as mesangial proliferation can be associated with red blood cell (RBC) casts yet would not require immunosuppressant treatment, whereas proliferative disease can be seen in the setting of a bland sediment [21]. Evaluation of the urine sediment alone is insufficient to determine whether kidney disease is present. A better approach is demonstrated in the British Isles Lupus Assessment Group (BILAG), European Consensus Lupus Activity Measure, and Systemic Lupus Activity Measure-Revised renal subscales, which consider the urine sediment in conjunction with eGFR and proteinuria. As pointed out by the American College of Rheumatology, before treatment decisions are placed solely on the urine sediment, reproducibility needs to be demonstrated [21].

The optimal renal subscale is one that is sensitive to change, whether improvement or deterioration. Both the magnitude and the presence of change are notable. This is best captured with serial measurement; therefore, development of a scale that is easy to administer and score is preferred. The requirement for formal training or complicated scoring will impair utility in clinical use.

## Renal endpoints in clinical trials of lupus nephritis: past and present

### Completed trials

Glucocorticoids and intermittent intravenous cyclophosphamide (IVC) have long been considered the standard induction agents to treat LN. Steinberg and Decker [22] reported the success of cyclophosphamide when compared with azathioprine (AZA) or placebo for inducing remission over the course of a 10-week period in a randomized trial of 38 patients during the mid-1970s. Patients were evaluated with respect to change in creatinine clearance, proteinuria, and urine sediment [22]. Amongst the treatment regimens studied by Austin and colleagues [1], the authors reported reduced rates of ESKD in a sample of mostly Caucasian patients who received IVC and glucocorticoids when compared to glucocorticoid monotherapy. LN has remained a research focus in an effort to identify more effective and less toxic treatment strategies. Table 2 includes a summary of important randomized controlled trials for the treatment of LN from the last 15 years. Although these studies have influenced the care of patients, none has led to FDA approval for an agent in the treatment of LN. Studies have varied in inclusion of patients by race and ethnicity, geographic region, size, duration of follow-up, and chosen primary and secondary endpoints.

**Table 2:** **An overview of landmark randomized controlled trials of lupus nephritis treatments published since 2000**

**Reference**	**Study design**	**Number of patients**	**Follow-up, months**	**Intervention**	**Steroids**	**Endpoint**	**Conclusion**
Induction							
Chan *et al*. [24] (2000)	Randomized, single-center study	42	12	MMF (1 g BID) versus CYC (2.5 mg/kg)	Prednisolone 0.8 mg/kg followed by taper, maintenance dose 10 mg/day	• Complete remission = Upr <0.3 g/day, with normal sediment, normal albumin, and SCr and CrCl ≤15% above baseline	Rate of remission similar between groups
						• Partial remission = Upr ≥0.3 and <2.9 g/day, albumin ≥3.0 g/dL, and stable kidney function	
						• Treatment failure = Upr ≥3.0 g/day, or Upr <3.0 with serum albumin <3.0 g/dL, SCr that has increased >0.6 mg/dL, or CrCl >15% above baseline	
Houssiau *et al*. [48] (2002)	Randomized non-inferiority, multicenter	90	41	High CYC (monthly pulses, dose adjusted based on WBC) versus low-dose CYC (500 mg every 2 weeks)	Methylpred three times followed by prednisolone taper, maintenance dose 5 to 7.5 mg/day	• Treatment failure = one of the following:	Similar treatment failure rates between groups
						○ Absence of a primary response after 6 months	
						○ Occurrence of glucocorticoid resistant flare	
						○ Doubling of serum creatinine	
						○ Lack of improvement in kidney function if dysfunction present at baseline	
Ginzler *et al*. [27] (2005)	Randomized, open-label, non-inferiority	140	6	Oral MMF daily (up to 3 g/day) versus monthly CYC (up to 1.0 g/m^2^)	Glucocorticoids 1 mg/kg per day followed by taper at clinician’s discretion	• Complete remission: return to within 10% of normal values for creatinine, proteinuria, and urine sediment	MMF was superior to CYC for induction
Appel *et al*. [25] (2009)	Randomized controlled, superiority trial	370	6	MMF (3 g/day) versus IV CYC (0.5 to 1.0 g/m^2^)	Glucocorticoids 60 mg followed by taper	• Response defined as:	Overall response rate the same in MMF and CYC groups
						○ Decrease in UPCR to <3 from a 24-hour collection in patients with baseline UPCR >3	
						○ Decrease in UPCR of >50% if sub-nephrotic at baseline	
						○ Stabilization (±25%) or improvement in serum creatinine	
Rovin *et al*. [28] (2012)	Randomized, placebo-controlled, multicenter	144	12	Addition of ritxumab versus placebo to MMF and steroids	Methylpred 1 g two times peri-study drug doses	• Complete renal response = normal SCr (if abnormal at baseline), inactive sediment, or UPCR <0.5	Response rates similar among groups
						• Partial renal response = SCr ≤115% of baseline, RBCs/hpf ≤50% above baseline, no RBC casts, and at least 50% decrease in UPCR or to <1.0 (if baseline was ≤3.0) or to ≤3.0 (if baseline was >3.0)	
						• No response = did not meet criteria for complete or partial response, terminated study early, or missing data limited ability to assess	
Furie *et al*. [26] (2014)	Randomized, phase II/III multicenter, double-blind study	298	12	Standard dose abatacept, high-dose abatacept, or placebo	Protocol defined steroid (and MMF) dosing	• Complete response = eGFR ≥90% of screening or pre-flare value, UPCR <0.26, inactive urinary sediment	Time to achievement of complete response was similar in all arms.
ACCESS trial group, 2014 [33]	Randomized double blind, double-blind, placebo-controlled	134	6 and 12 weeks	Euro-Lupus CYC with abatacept versus placebo	Methyl pred x3 followed by taper	Proportion of subjects achieving complete response at 24 weeks defined as:	No difference with abatacept
						• Kidney function: stable or improved eGFR	
						• Proteinuria: UPCR <0.5	
						• Urine sediment: not included	
						• Corticosteroid dose: tapered to ≤10 mg daily	
Maintenance							
Contreras *et al*. [29] (2004)	Single-center, randomized open-label trial	60	72	IV CYC (0.5 to 1.0 g/m^2^ every 3 months) or AZA 1 to 3 mg/kg per day or MMF (500 to 3,000 mg/day)	Glucocorticoids up to 0.5 mg/kg per day	• Patient survival	Patient survival was significantly better in AZA compared with CYC, and renal survival was similar in all groups.
						• Renal survival, defined as sustained increase in SCr to at least two times the lowest level achieved during induction, need for RRT or transplant	
Houssiau *et al*. [49] (2010)	Randomized trial	105	48	AZA (target 2 mg/kg per day) or MMF (target 2 g/day)	Methylpred three times followed by taper	• Time to renal flare = nephrotic syndrome, ≥33% increase in serum creatinine within 1 month, threefold increase in 24-hour proteinuria with hematuria, ≥ 33% reduction in C3 within 3 months	Fewer flares with AZA but failed to show superiority
Dooley *et al*. [30] (2011)	Randomized double-blind, double-dummy, multicenter	227	36	MMF (1 g BID) versus AZA (2 mg/kg daily)	Glucocorticoids 10 mg/day	• Time to treatment failure = time until the first event (death, ESKD, doubling of SCr, renal flare, or need for rescue therapy)	MMF superior to AZA

ACCESS, Abatacept and Cyclophosphamide Combination: Efficacy and Safety Study; AZA, azathioprine; BID, twice a day; CrCl, creatinine clearance; CYC, cystatin C; eGFR, estimated glomerular filtration rate; ESKD, end-stage kidney disease; hpf, high-power field; IV, intravenous; Methylpred, methylprednisolone; MMF, mycophenolate mofetil; RBC, red blood cell; RRT, renal replacement therapy; SCr, serum creatinine; UPCR, urinary protein-to-creatinine ratio; Upr, urinary protein excretion; WBC, white blood cell.

Studies can be divided into two types: studies of induction or maintenance of remission. An induction trial compares two treatments with respect to efficacy in achieving disease remission, whereas maintenance studies compare therapies with respect to limiting the frequency of flares [23]. Common endpoints in an induction trial are measures of disease activity. As demonstrated in Table 2, there is no standardized definition of ‘complete remission’. The definitions for remission of proteinuria vary from less than 0.3 g/day [24] to less than 3 g/day [25] to an improvement of more than 50% [25]. In studies that have included a measurement of eGFR, definitions of remission differ from comparisons made to the baseline value [24-26] versus comparisons made to normal values [27,28]. Trials of maintenance therapy focus on ‘treatment failure’ as the primary endpoint. These studies tend to be of longer duration, which affords them the opportunity to invoke hard endpoints such as patient survival, the need for renal replacement therapy, the occurrence of flare, or progressive kidney disease [29,30].

The trial that assessed efficacy and safety of adding abatacept to mycophenolate mofetil (MMF) highlights the need to define endpoints carefully [26]. The definition of ‘complete response’ chosen by those investigators was likely too restrictive as it included a composite measure that required maintenance of eGFR, minimal proteinuria, and inactive urinary sediment over the 52-week treatment period. This may be one reason why the response rate among all participants was much lower than expected [31,32].

It is unethical to deny study participants effective treatment, and therefore investigators must decide on a steroid dosing strategy in the trial design. As the examples in Table 2 illustrate, defining the dose and type of glucocorticoid to be used for induction is important but not standardized across trials. In addition, clinical trials must provide instructions for a taper and specify how to treat a flare to avoid confounding due to different cumulative steroid exposure between groups. Clear guidance on the use of medications, especially non-steroidal anti-inflammatory drugs (NSAIDs), angiotensin-converting enzyme inhibitors, or angiotensin receptor blockers, is also an essential component of trial design for LN.

The studies done to determine efficacy of MMF for remission of LN illustrate the distinction between superiority and non-inferiority trials. The studies by Chan and colleagues [24] and Ginzler and colleagues [27] were non-inferiority trials. Based on the success of MMF in these trials, the Aspreva Lupus Management Study Group trial was designed as a superiority trial, and numerous sites around the globe participated [25,31]. Comparable rates of patients responded to treatment in the two arms; however, MMF failed to demonstrate superiority, and therefore this was considered a negative trial.

### Ongoing clinical trials

Four major clinical trials to attempt to improve treatment of LN have recently been completed or are under way (Table 3). These include the following:

1. The ACCESS trial (Abatacept and Cyclophosphamide Combination: Efficacy and Safety Study), sponsored by the National Institute of Allergy and Infectious Diseases through the Immune Tolerance Network, assesses the efficacy of abatacept (a fusion protein composed of the Fc region of IgG1 fused to the extracellular domain of CTLA-4 that prevents T-cell activation) versus placebo in the treatment of proliferative LN (class III or IV with/without class V) with background therapy of Eurolupus IVC (500 mg IVC every 2 weeks for six doses) followed by maintenance with AZA [33,34].
2. The ALLURE (Advancing Leading-Edge Lupus Research) trial also assesses the efficacy of abatacept with background therapy of MMF.
3. The BLISS-LN (Belimumab International Lupus Nephritis Study) trial assesses the efficacy of belimumab—a human monoclonal antibody that inhibits the B-cell survival factor called B-cell activating factor (BAFF; also known as B-lymphocyte stimulator or BLyS) to prevent B-cell survival—with background therapy of Eurolupus IVC or MMF per investigator choice followed by MMF maintenance.
4. The ATLAS (Adjuvant Tamoxifen: Longer Against Shorter) trial assesses the efficacy of BIIB023—a humanized monoclonal antibody that inhibits tumor necrosis factor-related weak inducer of apoptosis (TWEAK) to reduce tissue inflammation—with background therapy of MMF.

**Table 3:** **An overview of major randomized controlled trials of lupus nephritis treatments currently in progress and their response criteria**

**ClinicalTrials. gov identifier**	**Sponsor**	**Study name**	**Study design**	**Lupus nephritis class**	**Intervention**	**Background therapy**	**Primary outcome**	**Patient enrollment goal, number**	**Sites, number**	**International or US only**	**Estimated completion date**
NCT01714817	Bristol-Myers Squibb	ALLURE	Phase 3, randomized, double-blind, placebo-controlled	III or IV	Abatacept versus placebo	Corticosteroids + MMF	Proportion of subjects achieving complete renal response at 52 weeks defined as:	400	98	International	July 2017
							1. Kidney function: eGFR normal or no less than 85% baseline				
							2. Proteinuria: UPC ratio <0.5				
							3. Urine sediment: no cellular casts				
							4. Corticosteroid dose: <11 mg daily for at least 28 days				
NCT01639339	Human Genome Sciences Inc., a GSK Company	BLISS-LN	Phase 3, randomized, double-blind, placebo-controlled	III or IV and coexisting V if present	Belimumab versus placebo	Corticosteroids + CYC for induction therapy	Number of participants with complete renal response at 104 weeks defined as:	464	102	International	February 2017
							1. Kidney function: eGFR no more than 10% below pre-flare value or normal				
						-AZA for maintenance OR High-dose steroids + MMF for induction therapy					
						-MMF for maintenance	2. Proteinuria: UPC ratio <0.5				
							3. Urine sediment: inactive (<5 RBCs/WBCs, no casts)				
							4. No rescue therapy				
	Biogen IDEC	ATLAS	Randomized, double-blind, placebo-controlled	III or IV and coexisting V if present	BIIB023 (anti-TWEAK) at high or low dose versus placebo	Corticosteroids + MMF	Proportion of subjects who achieve renal response (complete or partial) at 52 weeks	300	123	International	September 2016
NCT02141672	Aurinia	AURA-LV	Randomized, double-blind, placebo-controlled	III, IV and/or V	Voclosporin at high or low dose versus placebo	Corticosteroids + MMF	Number of subjects who achieve complete remission at 24 weeks defined as:	222	56	Inter-national	December 2016
							1. No confirmed decrease from baseline in eGFR of ≥20%				
							2. Proteinuria: UPC ratio <0.5				

ALLURE, Advancing Leading-Edge Lupus Research; ATLAS, Adjuvant Tamoxifen: AURA-LV, Aurinia Urinary Protein Reduction Active – Lupus with Voclosporin; Longer Against Shorter; AZA, azathioprine; BLISS-LN, Belimumab International Lupus Nephritis Study; CYC, cystatin C; eGFR, estimated glomerular filtration rate; MMF, mycophenolate mofetil; RBC, red blood cell; TWEAK, tumor necrosis factor-related weak inducer of apoptosis; UPC, urine protein-to-creatinine; WBC, white blood cell.

All four clinical trials use glucocorticoids as standard-of-care therapy. The ACCESS trial and BLISS-LN trial allow IVC remission induction therapy as another option. The ACCESS trial has completed 1-year follow-up data collection on the 134 participants, and the interim data have been published as abstracts [35]. The remaining three clinical trials are led by pharmaceutical companies and are attempting to recruit hundreds of patients from many national and international sites.

Each trial is designed with a primary, composite endpoint of complete renal response; however the criterion used in the endpoint definition varies across studies. Aside from the ACCESS trial, each study includes microscopic review of the urine sediment, namely looking for RBC casts, as a part of the composite endpoint. This may be questioned upon trial completion given the variability in an investigator’s ability to reliably and uniformly assess the sediment at each site. While a multi-faceted definition of complete renal response reflects the approach used in clinical care, it is likely difficult to achieve in a research setting, especially in a diverse group of patients recruited from centers around the world.

## Surrogate endpoints in chronic kidney disease trials

Clinical trials should be centered on improving outcomes that matter to patients. In the case of LN, many would agree that the prevention of the need for renal replacement therapy is the clinical endpoint of most concern. However, clinical trials often cannot afford to have as many years of follow-up as the early study by Austin and colleagues. As a result, many clinical trials are designed around surrogate endpoints. The National Institutes of Health defines a surrogate endpoint as a ‘biomarker intended to substitute for a clinical endpoint’; that is, a surrogate endpoint is a marker of a treatment effect that may correlate completely with a real clinical endpoint, but this relationship does not always hold true [36]. Surrogate endpoints are selected on the basis of their ability to predict the effect of a treatment on the clinical endpoint of interest [37,38]. The FDA allows for initial drug approval based on studies using surrogate endpoints with the caveat that post-marketing surveillance be performed to define long-term effects [39].

The development of novel agents for the treatment of CKD, from any cause, has been plagued by the need to identify appropriate surrogate endpoints. In diabetic nephropathy, the initial approvals for angiotensin-converting enzyme inhibitors and angiotensin receptor blockers relied on a doubling of serum creatinine, ESKD, or death as endpoints. Approval of newer agents to treat diabetic nephropathy, like that of agents to treat LN, has stalled while appropriate surrogate endpoints are defined [40]. Fortunately, the FDA and the American Society of Nephrology recently founded a public and private partnership of all stakeholders that is called the Kidney Health Initiative, which likely will facilitate development of appropriate surrogate endpoints in LN and other forms of kidney disease [41].

Surrogate endpoints commonly used in clinical trials of CKD include eGFR and proteinuria. Stevens and colleagues [37] published a thorough review of surrogate endpoints in trials of kidney disease several years ago, and we will summarize that review here. By definition, there must be a decrease in GFR for a patient to develop kidney failure, making substantial changes in GFR a reasonable intermediate endpoint for ESKD. However, changes in GFR are sometimes not appreciated in the early stages of kidney disease, and in the case of slowly progressive kidney disease, this may not be a useful endpoint if follow-up is not long enough. eGFR is considered to be a reflection of the number of functioning nephrons; however, glomerular hyperfiltration, glomerular hypertension, kidney perfusion, pregnancy, and medications including but not limited to NSAIDs all influence GFR and may interfere with interpretation [37].

The majority of patients with LN are women, who often have lower muscle mass and thus lower serum creatinine values and may be obese because of steroid exposure. Many clinical trials focus on patients with relatively preserved renal function. The current methods for estimating GFR are less precise with eGFR of more than 60 mL/min per m^2^. The search for a method less affected by weight and muscle mass to more precisely assess change in eGFR would strengthen this criterion as an outcome variable. Recent research assessing GFR employing cystatin C or iohexol dissipation in young diabetic, heart transplant patients or the general population may prove more helpful [42,43].

The power of a slope-based analysis can be jeopardized if the rate of underlying disease progression is not uniform over time or stage of disease. The use of a time-to-event analysis with a composite endpoint made up of objective endpoints such as the need for dialysis, a designated reduction in GFR, and an increase in serum creatinine helps circumvent some of these concerns [37].

Proteinuria has also been used as a surrogate endpoint because it correlates well with GFR and has been implicated in the pathogenesis of CKD. It is an attractive choice because, following an intervention, the change in proteinuria is often earlier and larger than the observed change in GFR. It might be useful therefore in slowly progressive or early stages of disease. The presence of or a change in proteinuria, unlike GFR, is not a mandatory intermediary in the development of kidney failure. As a result, proteinuria must be validated as a surrogate endpoint and this has yet to be done [37]. Complicating the search for surrogate markers are recent reports of patients with LN undergoing protocol repeat renal biopsy at defined time points regardless of clinical status. These have shown a significant discordance between complete or partial remissions defined by the measures above and histologic remissions. These results raise concern that repeat renal biopsy protocols may be required to define surrogate outcome measures for LN [44].

## Moving forward

Just as lupus is a multisystem disease, the design and execution of trials should be multidisciplinary. Input from nephrologists and rheumatologists who routinely care for patients with LN will help ensure that appropriate inclusion/exclusion criteria are chosen and that the selected primary and secondary endpoints are clinically meaningful. Although treatment options in LN have stalled, much can be learned from the trials that have been completed.

Time and again, we have seen success in early studies that was not replicated in multicenter, randomized, placebo-controlled trials. Investigators are charged with demonstrating that the addition of a study drug is more effective than usual care with steroids and current cytotoxic agents with respect to controlling disease, avoiding relapses, or lessening drug toxicity [23]. Given the many side effects associated with steroids, trials typically use and should continue to use a step-down design. This involves starting two agents simultaneously (for example, MMF and prednisone) and then reducing the dose of one agent (in this case, steroids) [23]. A treatment with similar efficacy that allows for steroid reduction would be a favorable option to clinicians and patients. Reduction in steroid dose is also an important endpoint that is not often used in clinical trials of LN.

The endpoints that we, as treating nephrologists and rheumatologists, favor for future clinical trials are composite endpoints that include assessment of GFR and proteinuria. For complete response, we favor proteinuria of less than 0.3 g, regardless of the starting point. The measure of GFR should account for change rather than a static arbitrary value. In patients with or without clinical response, a repeat kidney biopsy would provide definitive evidence of histologic response, including remission, and help validate the chosen surrogate endpoints. Just as a reduction in GFR is an inherent feature of kidney injury, persistent histologic evidence of proliferative nephritis signifies ongoing active kidney injury. GFR and proteinuria do not necessarily change as quickly as one another nor as quickly as the histology in a glomerulus. Therefore, to ensure that follow-up is long enough to permit change, clinical trials should include at least 12 (and, ideally, 24) months of follow-up. Table 4 includes a summary of our suggested endpoints in LN trials.

**Table 4:** **Take home points**

**1.**	Kidney disease activity rating scales in systemic lupus erythematosus should include the following:
	•A measure of estimated glomerular filtration rate (eGFR)
	◦Using consensus-based equation
	•A measure of proteinuria
	◦Using 24-hour collection
	•Analysis of urine sediment only in conjunction with above
**2.**	Guidelines for future trials of lupus nephritis:
	•Complete response should be a composite endpoint of the following:
	◦Proteinuria of less than 0.3 g/day
	◦eGFR:
	▪Stable (if more than 60 mL/min per m^2^ at baseline) or
	▪Improvement of at least 50% (if less than 60 mL/min per m^2^ at baseline)
	◦Consider repeat biopsy in patients meeting criteria for response or non-response
	◦Reduction or discontinuation of glucocorticoids
	•Treatment failure should be a composite of the following:
	◦Need for renal replacement therapy or transplant
	◦Persistent doubling of serum creatinine
	◦eGFR decrease by at least 50%
	◦Renal flare requiring treatment
	◦Death
	•Design should require 12 to 24 months of follow-up.
	•Detailed guidance on steroid dosing should be provided.

All study participants must be offered effective treatment such as steroids, anti-malarial agents, angiotensin-converting enzyme inhibitors or angiotensin receptor blockers, and HMG-CoA reductase inhibitors. Patients entering a trial should receive the same regimen of glucocorticoids with a standard tapering schedule [23]. For example, the abatacept trial did not restrict steroid dosing, and the analysis observed a trend of higher mean prednisone dose among the placebo group, confounding interpretation of the data [26].

Lupus has diverse manifestations, and this is likely due to numerous subtypes of disease. Studying new agents in a more homogeneous patient sample may increase the yield of positive findings. The research into rituximab illustrates this point. The earlier uncontrolled trials were of patients who failed initial therapy with cyclophosphamide or MMF, whereas the LUNAR (Lupus Nephritis Assessment with Rituximab) project enrolled patients receiving initial treatment [31]. It must also be realized, though, that using more inclusion and exclusion criteria must be weighed against the difficulties of patient recruitment.

## Conclusions

The diverse manifestations of SLE pose challenges in the design of clinical trials. To capture disease activity, various disease activity indices have been developed. Thesevary in length and complexity, and many have renal subscales. The renal subscales, particularly in the SLEDAI-2 K (Systemic Lupus Erythematosus Disease Activity Index 2000) and BILAG tools, measure clinically meaningful parameters of kidney involvement, namely eGFR and proteinuria (Table 4). It is important that the best method to define eGFR and proteinuria in LN be identified and then adopted into clinical care and trial design. To advance the knowledge of how to treat LN, well-designed clinical trials informed by prior randomized controlled trials are needed. Clinical trials should have thoughtful inclusion and exclusion criteria, pre-specified dosing parameters for glucocorticoids and other medications, and well-designed endpoints.

## Note

This article is part of the series ‘*Measuring meaningful change in lupus clinical trials’*, edited by Matthew Liang and Chan-Bum Choi. Other articles in this series can be found at http://arthritis-research.com/series/trials

## Abbreviations

ACCESS: Abatacept and Cyclophosphamide Combination: Efficacy and Safety Study
AZA: azathioprine
BILAG: British Isles Lupus Assessment Group
BLISS-LN: Belimumab International Lupus Nephritis Study
CKD: chronic kidney disease
eGFR: estimated glomerular filtration rate
ESKD: end-stage kidney disease
FDA: US Food and Drug Administration
IVC: intravenous cyclophosphamide
LN: lupus nephritis
MMF: mycophenolate mofetil
NSAID: non-steroidal anti-inflammatory drug
RBC: red blood cell
SLE: systemic lupus erythematosus
